# Association of Brain Natriuretic Peptide With Mortality in Exceptionally Long-Lived Families

**DOI:** 10.1093/geroni/igaa057.681

**Published:** 2020-12-16

**Authors:** Allison Kuipers, Robert Boudreau, Mary Feitosa, Angeline Galvin, Bharat Thygarajan, Joseph Zmuda

**Affiliations:** 1 University of Pittsburgh, Pittsburgh, Pennsylvania, United States; 2 Washington University School of Medicine, St Louis, Missouri, United States; 3 University of Southern Denmark, Odense C, Midtjylland, Denmark; 4 University of Minnesota, Minneapolis, Minnesota, United States

## Abstract

Natriuretic peptides are produced within the heart and released in response to increased chamber wall tension and heart failure (HF). N-Terminal prohormone Brain Natriuretic Peptide (NT-proBNP) is a specific natriuretic peptide commonly assayed in persons at risk for HF. In these individuals, NT-proBNP is associated with future disease prognosis and mortality. However, its association with mortality among healthy older adults remains unknown. Therefore, we determined the association of NT-proBNP with all-cause mortality over a median follow-up of 10 years in 3253 individuals free from HF at baseline in the Long Life Family Study, a study of families recruited for exceptional longevity. We performed cox proportional hazards analysis (coxme in R) for time-to event (mortality), adjusted for field center, familial relatedness, age, sex, education, smoking, alcohol, physical activity, BMI, diabetes, hypertension, and cancer. In addition, we performed secondary analyses among individuals (N=2457) within the normal NT-proBNP limits at baseline (<125pg/ml aged <75 years; <450pg/ml aged ≥75 years). Overall, individuals were aged 32-110 years (median 67 years; 44% male), had mean NT-proBNP of 318.5 pg/ml (median 91.0 pg/ml) and 1066 individuals (33%) died over the follow-up period. After adjustment, each 1 SD greater baseline NT-proBNP was associated with a 1.30-times increased hazard of mortality (95% CI: 1.24-1.36; P<0.0001). Results were similar in individuals with normal baseline NT-proBNP (HR: 1.21; 95% CI: 1.11-1.32; P<0.0001). These results suggest that NT-proBNP is a strong and specific biomarker for mortality in older adults independent of current health status, even in those with clinically-defined normal NT-proBNP.

